# *Meta* synthetic biology: controlling the evolution of engineered living systems

**DOI:** 10.1111/1751-7915.13350

**Published:** 2018-12-12

**Authors:** Morten H. H. Nørholm

**Affiliations:** ^1^ Microbial Evolution and Synthetic Biology Group Novo Nordisk Foundation Center for Biosustainability Technical University of Denmark 2800 Kgs. Lyngby Denmark

## Abstract

A major aim of synthetic biology is the design of robust living systems for real‐world applications. In seemingly contrast, evolution changes the living, exploring new survival strategies in response to environmental challenges. How do we cope with this paradox? Can we control or even exploit the molecular mechanisms of evolution for biotechnological and biosustainable innovation and will the principles of engineering lead to fundamental insights in evolutionary biology? A merger of synthetic biology with experimental evolution is occurring and it will radically accelerate the development of these scientific disciplines.

The second half of the 20th century saw the birth and maturation of molecular biology as a scientific discipline, reaching a major milestone with the release of the human genome sequence in 2001. In the recent two decades, synthetic biology has emerged as the next‐generation molecular biology with emphasis on engineering concepts such as robustness, standardization, design‐build‐test and application of living systems.

In parallel, the next‐generation DNA sequencing (NGS) technologies have led to a renaissance for experimental evolution. For example, Richard Lenski′s 30 ‐year‐old and ongoing bacterial ‘Long Term Evolution Experiment’ is providing fundamental insights into evolutionary mechanisms such as the development of new nutrient utilization phenotypes, evolution of co‐existing communities and the effect and development of different mutation rates (Lenski, 2017). Other laboratories have focused on more applied aspects of ‘Adaptive Laboratory Evolution’ such as cell factory tolerance development to various types of stress in connection with industrial process conditions (Portnoy *et al*., 2011). These endeavours beautifully showcase a scientific field moving from being mainly descriptive towards hypothesis‐driven and experimentally driven research, leading to a paradigm shift in the understanding of the underlying processes followed by a bloom in new technologies and applications.

The transition from molecular to synthetic biology – from *reading* to *writing* biology – is happening at a rapid pace, driven by paradigm‐shifting technologies such as PCR and CRISPR, but biology is complex and even the simplest designs explore only a fraction of the infinite solution space we call nature. Worse, once reengineered biological systems work to our satisfaction, robustness over longer timescales appears as a huge challenge. This reflects not simply the inherent complexity in nature, but rather that *change* is something fundamental to the success of the living *–* perhaps best exemplified with the prevalence of ageing and death in nature: immortality is not a favourably trait.

The change and fitness of the modified biological system is at the core of Darwinian evolution, but in contrast to the early view on change as an entirely random process, the molecular biology era has provided compelling evidence for specific chemical reactions and molecular mechanisms that highly impact the physical nature of mutations and the rate of their appearance. Molecular biologists have long been able to manipulate the rate of mutations in living cells by e.g. deleting DNA repair systems, reducing DNA replication accuracy or introducing DNA modifying enzymes. More recently, these global mutator mechanisms have been reengineered for higher accuracy, enabling mutagenesis in selected regions of genomes, for example by using CRISPR to target an error‐prone polymerase or specific DNA modifying enzymes to highly specific locations (Komor *et al*., 2016; Halperin *et al*., 2018).

Manipulation of different repair mechanisms can change the rate of specific mutations, but different environmental conditions similarly affect the evolutionary chemistry without necessarily changing the rates: In some ageing bacterial colonies, G‐>T mutations occur at orders of magnitude more frequently than the other five possible single nucleotide mutations in DNA [Fig. 1A, (Hall, 1991)]. In line with these findings, by applying a combination of genome re‐sequencing with experimental evolution, we recently found evidence for the prevalence of G‐>T mutations in ageing *Escherichia coli* colonies and observed that they occurred highly dominantly on the transcribed strand in genes (Sekowska *et al*., 2016). Under these conditions, suddenly the mutational space is considerably reduced to predominantly one type (i.e. G‐>T on the transcribed strand) out of a total of 12 possible single nucleotide mutations (Fig. 1B) and only 19 possible amino acid changes out of the theoretical 380 (Fig. 1C). Similarly, studies from the Ferenci group showed highly distinct mutational profiles under different nutritional stresses (Maharjan and Ferenci, 2017).

**Figure 1:**
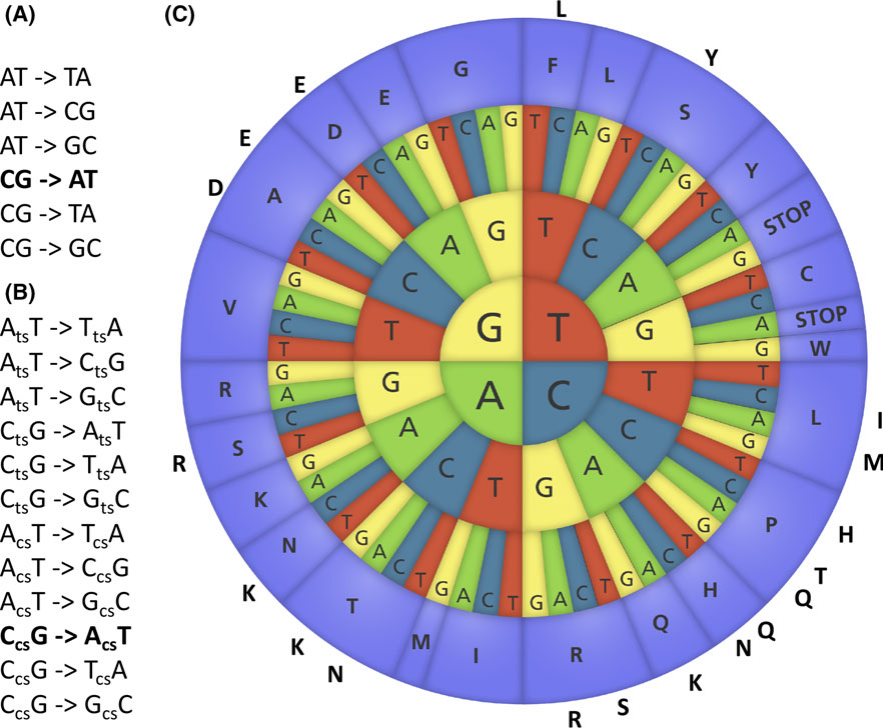
The mutational landscape is affected by environmental conditions.A. Six different single nucleotide mutations are possible in DNA. G‐>T mutations (bold) are known to dominate in ageing bacterial colonies likely due to oxidative stress (Sekowska *et al*., 2016).B. When taking strand bias into account, G‐>T mutations are only one in theoretically 12 different types of single nucleotide mutations (cs, coding strand; ts, transcribed strand). C. G‐>T mutations on the transcribed strand explores a very limited set of 19 out of a possible 20 × 19 = 380 different amino acid changes. Genetic code illustration modified from http://www.yourgenome.org/under the Creative commons license.

As a result, under specific conditions and because the universal genetic code is degenerate, it is possible to influence how genes or whole genomes evolve without changing the immediate phenotypic characteristics, simply by changing the nucleotide composition. When G‐>T mutations dominate, reducing the use of G should limit the available nucleotides for mutagenesis, right? It is tempting to speculate that this potential has been realized in nature already and that this may have shaped the genetic code and parameters such as the GC/AT ratio of genomes. As we explore the mechanisms of evolution further, we may increasingly realize how such regulatory regimes of specific repair mechanisms reflect the physical nature of mutations under specific environmental conditions.

In summary, at first sight, there is a sharp contrast between rational synthetic biology and random evolution. However, tinkering and prototyping are often integrated in design‐build‐test engineering workflows and the way evolution occurs in nature sometimes appears remarkably rational – as the quasi‐Lamarckian CRISPR mechanisms have reminded us recently. My main claim in this short piece is that we are experiencing a growing trend of merging bioengineering with experimental evolution on several levels: the nature and rate of mutations can be manipulated both globally (the genome) and locally (individual genes or sections of the genome) in living cells, and interdisciplinary cross‐fertilization occurs as we start *engineering the evolvability* and *evolving the engineered*.

As our synthetic biology toolbox become increasingly sophisticated and our fundamental insights into evolutionary mechanisms are fuelled by e.g. NGS and other omics technologies, we may end up synthesizing entire living systems bottom‐up and fine‐tune their performance using controlled evolution. In the near future, we will surely create microbes that are genetically hyper stable for robust performance in bioreactors, but what about microbiome therapies that will evolve in the gut and become personalized to fit the host genetics perfectly?

With this year's Nobel Prize to Frances Arnold for her work on directed evolution of enzymes, perhaps we are only seeing the beginning of evolutionary applications. Soon it will be mainstream to not only engineer the living, but to engineer how the living changes. The term Meta Synthetic Biology could be used to describe the added layer of temporal development and evolution of synthetic biology systems – and to describe the merger of synthetic biology with experimental evolution – in my view two of the most exciting contemporary scientific disciplines.
